# Behavioural Adaptation of a Bird from Transient Wetland Specialist to an Urban Resident

**DOI:** 10.1371/journal.pone.0050006

**Published:** 2012-11-21

**Authors:** John Martin, Kris French, Richard Major

**Affiliations:** 1 Institute for Conservation Biology and Environmental Management, School of Biological Sciences, University of Wollongong, Wollongong, New South Wales, Australia; 2 Terrestrial Ecology, Australian Museum, Sydney, New South Wales, Australia; University of Utah, United States of America

## Abstract

Dramatic population increases of the native white ibis in urban areas have resulted in their classification as a nuisance species. In response to community and industry complaints, land managers have attempted to deter the growing population by destroying ibis nests and eggs over the last twenty years. However, our understanding of ibis ecology is poor and a question of particular importance for management is whether ibis show sufficient site fidelity to justify site-level management of nuisance populations. Ibis in non-urban areas have been observed to be highly transient and capable of moving hundreds of kilometres. In urban areas the population has been observed to vary seasonally, but at some sites ibis are always observed and are thought to be behaving as residents. To measure the level of site fidelity, we colour banded 93 adult ibis at an urban park and conducted 3-day surveys each fortnight over one year, then each quarter over four years. From the quarterly data, the first year resighting rate was 89% for females (*n* = 59) and 76% for males (*n* = 34); this decreased to 41% of females and 21% of males in the fourth year. Ibis are known to be highly mobile, and 70% of females and 77% of males were observed at additional sites within the surrounding region (up to 50 km distant). Our results indicate that a large proportion of ibis have chosen residency over transience both within the study site and across the broader urban region. Consequently the establishment of refuge breeding habitat should be a priority localised management may be effective at particular sites, but it is likely to have an impact across the broader population.

## Introduction

Understanding site fidelity is important for the conservation of rare species [1] and the management of introduced pest or nuisance native species [2], [3]. Site fidelity is likely to be affected by resource availability, which influences a species’ movements and behaviour [4]. The urban environment appears to have abundant habitat and foraging resources for a range of species and these resources are being utilised by both rare [5], [6], and pest species [7], [8]. Behavioural flexibility to novel food resources is a mechanism that assists species to colonise new habitats [9]. As a result of high resource availability, individuals adapting to urban habitats are predicted to alter their behaviour, influencing rates of immigration, emigration, survival and fecundity [10]. High resource levels in cities relative to natural habitats may cause some species to increase their population sizes.

The Australian white ibis (*Threskiornis molucca*), hereafter referred to as “ibis”, is considered a nuisance native species in urban areas because its population has increased and encroaches upon humans (see ‘Study species and site’) [11], [12]. In response, land managers have destroyed ibis nests and eggs to deter breeding and reduce recruitment [13]. Traditionally, large numbers of ibis were observed breeding within flooded inland wetlands, and movements of hundreds of kilometres were observed between these ephemeral resources [14], [15]. An assessment of ibis breeding across the State of Victoria between 1955 and 1980 identified 63 sites and concluded that site use, the number of birds and the timing of breeding were annually variable [15]. Breeding occurred in an average of 7.6±0.9 se of 25 years per site, with the amount of breeding varying from tens of birds up to ∼20 000 based on the data provided for 25 colonies [15]. This annual variability reflects the unpredictable Australian climate where there is strong selection for native species to evolve behavioural plasticity [16], [17]. Consequently ibis have been observed to breed sporadically at particular sites and at any time of the year as conditions allow [14], [15]. Long distance movements between unpredictable habitat is likely to be energetically costly and may have an impact on survival and fecundity [18], thus selection should favour site fidelity over transience where resources are consistently abundant [19], [20], such as in urban areas. Increased site fidelity has been found in urban habitats for some synanthropic species [7], [21], [22], but little information is available on site fidelity within urban ibis populations.

Site fidelity has been defined as the degree to which an animal returns to a specific site. Studies commonly assess site fidelity with respect to animals returning to breeding grounds [19], [23] or moving through migratory staging grounds [24], [25]. Ecologically, studies assessing site fidelity are questioning behavioural decision-making with respect to patch quality [18], [24], [26], and this question is being applied to resource availability in urban habitats [27], [28].

Understanding the site fidelity of ibis is important to inform both management actions aimed at resolving human-wildlife conflicts, and for conservation. The urban environment is thought to be providing a refuge for ibis [8] as the inland wetland habitat has been degraded from human modification of river flows [29], [30]. Ibis are long-lived (up to 26 years, [31]) and the breeding success of urban populations may be important for maintaining populations of this species at a continental scale, as only a few small natural breeding events have been documented over the last decade [32], [33]. In this study we assess the site fidelity of ibis to an urban park, defining high site fidelity as presence on greater than or equal to 75% of surveys within a calendar year.

As abundant foraging and habitat resources are consistentlypresent within the urban environment [7], in contrast to the ephemeral inland wetlands [16], [17], a high degree of site fidelity might be expected in ibis that occupy cities. The aims of this study are 1) to determine levels of site fidelity of male and female ibis, 2) to assess the extent of movements to foraging sites and breeding colonies within the study region, and 3) to measure fluctuations in the population at the study site over a 4-year period.

## Methods

### Ethics Statement

Bird capture, banding and surveys were conducted in accordance with the University of Wollongong Animal Care and Ethics Committee (AE05-15r8); Australian Museum Animal Care and Ethics Committee (08-05); New South Wales National Parks and Wildlife Service Scientific Licences (S12568, S12586) and an Australian Bird and Bat Banding Scheme research project (1209).

### Study Species and Site

Ibis are large wetland birds with mostly white plumage, a wingspan to 125 cm, a long down-curved bill (to 22 cm) and a black unfeathered head and neck (only in adults ≥3years) [34]. Up to 7000 adult ibis are known to occur within the Sydney region, Australia, throughout the year [12], and they are frequently observed in city parks. Centennial Park (33°53′′56′S, 151°14′′01′E), near the centre of Sydney, is a large urban park (186 hectare) containing ponds of varying size (26 ha total area), some of which contain small islands. Ibis are always present and nest on the islands and forage within the grass and pond habitats. In addition, people commonly feed the birds (e.g. bread) within the park, and scavenging occurs from bins and picnickers.

Ibis have the capacity to be highly mobile. During daily foraging, multiple movements of 10′s of kilometers between roosting and foraging resources have been recorded during radio telemetry [8]. Larger movements of 100’ s of kilometres have been inferred through the colonisation of distant breeding sites following the flooding of a wetland [14]. At the site scale the disappearance of a bird could be the result of a movement within the surrounding region (10’ s km) or outside the region (100 km’s). For ibis transient movements are likely to involve leaving an area for an extended period (e.g. months) and population surveys of the Sydney region have identified that adult ibis seasonally immigrate to the region and disperse following breeding [12]. This seasonal population increase, which is associated with breeding, fuels community complaints.

Ibis are considered to present a pest problem for four main reasons. Firstly, their population increase in urban areas over the last 30 years [12] has lead to the misconception that they are the African sacred ibis (*T. aethiopicus*) and should be managed as a non-native pest (as is the case in Europe and North America [35], [36]). Secondly, ibis are known to carry diseases and there is a concern that humans are at risk of infections from birds living in close proximity to humans [37]. Thirdly, people object to the smell, noise, defecation and scavenging of ibis in urban environments. Finally, the ingestion of an ibis by in a jet engine in 1995 led to the realization that ibis colonies near airports presented a major aviation hazard. During the late 1990’s the ibis population within the study site was reduced from a seasonal peak of over 1000 down to 400 ibis through the removal of nesting habitat and the destruction of nests [J. Cartmill pers. comm.]. No habitat or nest destruction has been implemented at the study site since 2005. However, the management of nesting ibis within urban parks, industrial land and residences is commonplace across the Sydney region to mitigate local complaints. Foraging by ibis within landfills is thought to be sustaining the large urban population and the extended breeding season [12], which cause the major issues for the general public.

### Field Surveys

Adult ibis were individually caught (*n* = 34 males and 59 females) within the park between July 2005 and April 2006 using a hand-held foot noose made of fishing line with bread as an attractant. Ibis were individually colour-banded with a combination of four bands on the tarsus and tibia [38]. The sex of each bird was determined based upon bill length (exposed culmen, [34]).

Surveys of the ibis population within the park were conducted over three consecutive days. During these surveys we aimed to re-sight banded ibis and count the total number of adults and juveniles in the park. A single observer traversed a route that covered all areas where ibis occurred (all but two of the surveys were conducted by the same observer). Surveys were conducted for three hours during the afternoon (∼3pm to ∼6pm, depending on day-length) when ibis were most abundant [39] using binoculars and a spotting scope. Between January 2006 and April 2007 fortnightly 3-day surveys (*n* = 32) were conducted to intensively assess site fidelity. Following this, 3-day surveys were conducted quarterly (Jan, Apr, July, Oct) until the end of 2009 (*n* = 16, including four from 2006). From March 2007 we also counted the number of active nests (defined as containing eggs, nestlings or under incubation) and from September 2007 we noted if the legs, and therefore colour bands, of adult ibis were unidentifiable due to obstructions (e.g. water, vegetation or nest material). Banded ibis were also resighted outside of the study site during monthly surveys of the Sydney region. These surveys were conducted over three consecutive days by a single observer and included the largest foraging and breeding locations as well as smaller sites (*n* = 54) where ibis occur in the Sydney region. At all sites, we recorded the number of adults, juveniles, nestlings and nests (for further detail see [12]).

### Statistical Analysis

The survival rate of colour-banded ibis and rates of temporary emigration and immigration were estimated for both sexes using the robust design [40] within Program MARK [41]. By incorporating ‘replicate’ surveys in the same survey period (i.e. surveys on three consecutive days) the robust design is able to produce estimates of the temporary emigration and immigration rates. The parameters of emigration and immigration allow for, and estimate, the likelihood of marked individuals moving in and out of the study location between survey periods (e.g. quarterly surveys). This in turn improves the survival estimation and our interpretation of site fidelity. The robust design estimates five parameters in each model (Table 1 & 2): survival rate (*φ*), emigration rate (*γ′′*), immigration rate (*γ′*), initial capture probability (p) and recapture probability (c). Each of these parameters was estimated in a range of models that included the effects of sex (g) and time (t). A period (.) represents a variable that is held constant e.g. the exclusion of time-specific effects. Model selection was based on Akaike’s Information Criterion [42].

**Table 1:** Best-fitting models of resighted banded ibis assessed using the robust design within Program MARK.

Model*	AICc	ΔAICc	Model Likelihood	Num. Par	Deviance
{*φ*(g,.), *γ′′*(g,.), *γ*′(g,.), p(g,.), c(g,.)}	5048	0	0.99967	127	7143
{*φ*(g,t), *γ′′*(g,.), *γ*′(g,.), p(g,.), c(g,.)}	5064	16	0.00029	151	7101
{*φ*(g,.), *γ′′*(g,.), *γ*′(g,.), p(g,.), c(g,t)}	5099	51	0.00004	173	7080

Surveys were conducted (a) fortnightly (*n* = 32) over 15-months.

Studies reporting site fidelity commonly provide a species-specific definition [18], [43]. For this study we have defined high site fidelity as birds observed on greater than or equal to 75% of the fortnightly (conducted over 15-months, *n* = 32) and quarterly surveys (*n* = 4 within a calendar year). Site fidelity was measured for each sex separately, by estimating the frequency with which male and female ibis were resighted within the study site. The percentage of surveys in which an individual was resighted was amalgamated into the percentile categories of 0, 1 to 25, 26 to 50, 51 to 75 and 76 to 100, for both the fortnightly and quarterly data. For the fortnightly surveys (*n* = 32) we calculated the frequency that males and females were resighted and measured comparative site fidelity with respect to sex using Chi-square analysis. We then looked at both the breeding (July to December) and the non-breeding (January to June) seasons [12], [39], [44] to determine whether sex specific differences in residency varied seasonally. For the quarterly surveys we calculated the frequency with which both male and female ibis were resighted in each year (2006 to 2009) and determined sex specific differences in the annual site fidelity, again using Chi-square analysis. We also used Chi-square analyses to determine whether male or female ibis were more likely to be resighted outside of the study area and whether they differed in their use of the major habitat categories of landfills, parks or breeding colonies.

The relationships between ibis abundance within the study site, the season (non-breeding, breeding) and the year (2006, 2007, 2008 and 2009) were assessed using a two-factor analysis of variance (ANOVA). Separate analyses were conducted for adults and juveniles. The breeding season of adult ibis was defined as July to December based on nesting records, but breeding has also been observed at low levels all year round [12], [44]. The analysis of juveniles was lagged by one month to account for the delay between egg laying and fledging. Annual variation in reproductive activity, as measured by counting the number of nests at the study site during three breeding seasons (2007, 2008, 2009), was compared with a single-factor ANOVA. The relationship between the number of unidentifiable adult ibis and season (non-breeding, breeding) was investigated using a single-factor ANOVA, as the resighting rate of banded birds may be reduced due to nest incubation (these data are a subset of the total population count assessed above). All data were tested for homogeneity of variance with Cochran’s test and, where necessary, log(*x*+1) transformations were used to stabilise variances. Significant interactions were identified with Tukey’s pairwise comparisons.

## Results

### Short-term Survival and Residency

The best fitting model for the 15-months of fortnightly surveys included a constant time parameter for survival, emigration, immigration and the capture and recapture probabilities. This model was superior to models that included separate parameters for each time interval (Table 1). Using this model, the survival rate for both females and males was estimated to be close to 100% (female = 0.99±0.005 se; male = 0.98±0.013) over the 15-months of fortnightly surveys. Because ibis are thought to be long-lived a high survival rate was expected. Of greater interest is the estimated probability of emigration, that is, an observed banded ibis not being present during the next survey. The model estimated that 17% (±0.021) of females and 21% (±0.031) of males would leave the study site between fortnightly surveys. Of the individuals that were not observed during a survey, the model estimated that 86% (±0.022) of females and 94% (±0.020) of males would not be resighted during the next survey. This means that unobserved ibis are unlikely to be resighted within the study site for an extended period.

**Figure 1:**
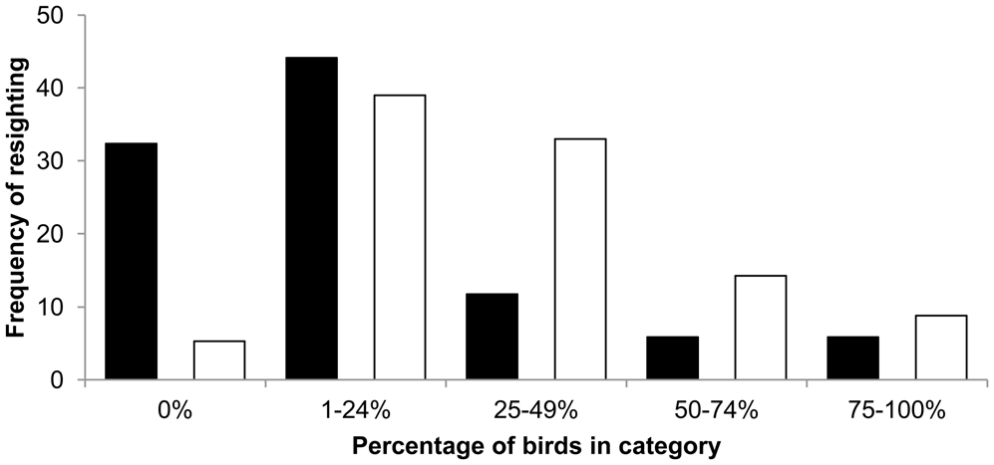
Residency of male (black, n = 34) and female (white, n = 59) ibis over 15-months from pooled fortnightly 3-day surveys (n = 32).

Corroborating the MARK analysis, the female resighting frequency was greater than that of males (χ_4_^2^ = 15.8, p = 0.003) indicating greater site fidelity over the 15-month fortnightly survey period (Fig. 1). A high level of site fidelity (i.e. resighted in greater than or equal to 75% of surveys) was observed for a small number of females (9% *n* = 59) and males (6% *n* = 34). The site fidelity of female ibis appeared to be greater than that of males during the non-breeding season (January to June) with 75% (*n* = 59) of females being resighted compared to 59% (*n* = 34) of males, but this difference was not statistically significant (χ_4_^2^ = 8.02, p = 0.09). Similarly, the site fidelity of females appeared to be greater than that of males during the breeding season (July to December) with 77% (*n* = 59) of females being resighted compared to 47% (*n* = 34) of males, but again, the difference was not statistically significant (χ_4_^2^ = 9.21, p = 0.056).

### Long-term Survival and Residency

The best fitting model for the 4-year quarterly surveys included a constant time parameter for survival, emigration, immigration and the capture and recapture probabilities. This model was superior to models that included separate parameters for each time interval (Table 2). Over the 4-year study a survival rate of 92% (±0.014) was estimated for female ibis and 87% (±0.028) for males by the best fitting model. Between quarterly surveys, the model estimated that 33% (±0.036) of females and 42% (±0.088) of males would emigrate from the study site. Of the individuals that were not observed during a survey, it was estimated that 66% (±0.048) of females and 74% (±0.075) of males would not be resighted during the subsequent survey.

**Table 2:** Best-fitting models of resighted banded ibis assessed using the robust design within Program MARK.

Model*	AICc	ΔAICc	Model Likelihood	Num. Par	Deviance
{*φ*(g,.), *γ′′*(g,.), *γ′*(g,.), p(g,t) = c(g,t)}	2348	0	0.9943	98	2871
{*φ*(g,t), *γ′′*g,.), *γ′*(g,.), p(g,.), c(g,.)}	2359	11	0.0050	67	2863
{*φ*(g,t), *γ′′*(g,t), *γ′*(g,.), p(g,.), c(g,.)}	2363	15	0.0007	133	2783

* The robust design estimates five parameters in each model: survival rate (*φ*), emigration rate (*γ”* ), immigration rate (*γ’* ), initial capture probability (p) and recapture probability (c). The symbols within parentheses indicate whether the parameter is held constant ‘.’ or whether separate parameters are include for each sex (g) or survey interval (t).

Surveys were conducted quarterly (*n* = 16) over 4-years.

**Figure 2:**
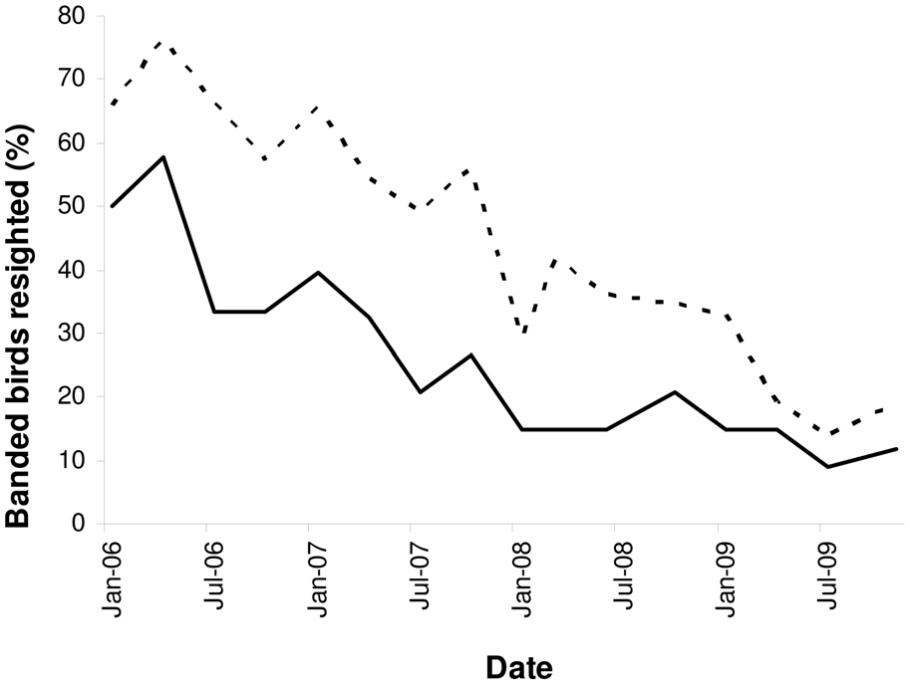
Resighting rate of colour-banded male (solid, n = 34) and female (dash, n = 59) ibis over 4-years from pooled quarterly 3-day surveys.

The number of banded ibis that were resighted in each survey decreased over the four years and there was little evidence of a seasonal effect on sex-based differences in site fidelity (Fig. 2). The number of resighted female and male ibis was not significantly different during 2006 (χ_4_^2^ = 8.39, p = 0.078) and 2009 (χ_4_^2^ = 5.22, p = 0.265); however, a significantly higher number of females were resighted in 2007 (χ_4_^2^ = 10.32, p = 0.035) and 2008 (χ_4_^2^ = 13.03, p = 0.011). A greater percentage of females displayed high site fidelity, defined as being observed in greater than or equal to 75% of surveys in each year. Thirty-seven percent (*n = *59) of females and 18% (*n = *34) of males showed high site fidelity in 2006, 47% of females and 21% of males in 2007, 26% of females and 15% of males in 2008 and 16% of females and 9% of males in 2009.

Throughout the study we were unable to determine whether every ibis was colour-banded due to occasional visual obstruction of their legs. In 2008 and 2009 the number of unidentifiable adult ibis within the population during a survey of the study site varied between a minimum of 2% and a maximum of 38% (*n* = 3 and *n* = 137 unidentifiable ibis, respectively). Unsurprisingly, the proportion of adult ibis with unidentifiable legs was significantly greater during the breeding season (July to December) when some birds were sitting on nests (*F*_1,22_ = 36.27, *P*<0.000).

### Population Size

**Figure 3:**
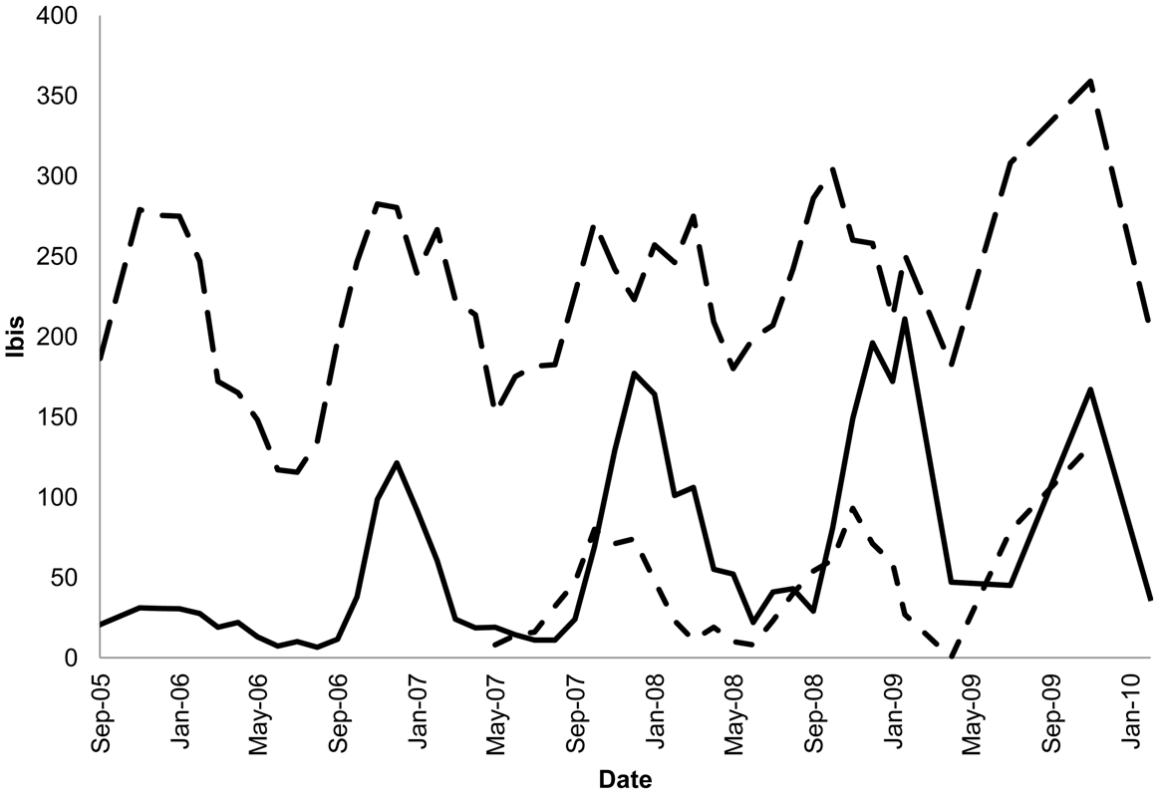
Ibis within Centennial Park showing the annual fluctuations associated with the breeding cycle for adults (long-dash), juveniles (solid) and nests (dash).

The number of adult ibis using the study site did not vary significantly between years (*F*_3,32_ = 2.4, *P* = 0.08, Fig. 3), but was greater in the breeding seasons than in the non-breeding seasons (*F*_1,32_ = 6.55, *P* = 0.015). There was no significant interaction between season and year, with the same seasonal fluctuations detected in each of the four years (*F*_3,32_ = 1.9, *P* = 0.15). During the breeding seasons (July to December) the number of nests observed increased over the three years (*F*_2,15_ = 6.39, *P* = 0.01) (Fig. 3). More nesting occurred during the 2009 breeding season compared with that of 2007 (Tukey pairwise, *P* = 0.009) and 2008 (Tukey pairwise, *P* = 0.058); levels of nesting in the latter two years were comparable (Tukey pairwise, *P* = 0.63). The number of juveniles differed between years (*F*_3,31_ = 4.18, *P* = 0.013, Fig. 3) with significant increases occurring during the study. Significantly higher numbers of juveniles were observed between August to January than February to July (*F*_1,31_ = 8.77, *P* = 0.006), and there was no significant interaction between season and year (*F*_3,31_ = 0.34, *P* = 0.80).

### Resightings Beyond the Study Site

Ibis banded in Centennial Park were commonly resighted in surveys across the Sydney region (for survey methods, see [12]), with both sexes equally likely to be resighted (χ_1_^2^ = 0.23, p = 0.63). We observed 70% (*n = *59) of colour-banded females at one or more of 32 different sites away from the study site and 77% of males (*n = *34) at one or more of 20 sites. Landfills were a major foraging resource with comparable usage by birds of each sex (χ_1_^2^ = 0.43, p = 0.51); we encountered 41% of females (*n* = 59) and 50% of males (*n* = 34) at least once at one or more of the five open landfills in the region. For example, 19% of females and 35% of males were observed at least once at a landfill 20 km to the north, and 24% of females and 18% of males were observed at least once at a landfill 35 km to the west of the study site. Other locations at which ibis were frequently seen in the Sydney region included parkland and breeding colonies. Parks provided similar resources to the study site yet only 36% of females and 29% of males were observed therein (χ_1_^2^ = 0.82, p = 0.36). Interestingly, at breeding colonies we observed twice as many males (29%) as females (14%) at least once, although this was not significant (χ_1_^2^ = 2.53, p = 0.11).

## Discussion

For species able to exploit them, urban environments have the attributes of abundant habitat and consistent resources [22], [45], [46]. The behaviour of species that have colonised urban environments has often been observed to change from that of their ‘natural’ behaviour [5]–[7], [47]. For example, studies of European Blackbirds have found that urban birds are more sedentary than rural birds [47], [48]. Approximately one quarter of the banded ibis in this study were observed to exhibit a high level of site fidelity in three of the four years, and given our inability to detect all banded birds in a survey, this figure represents the minimum estimate. This level of site fidelity observed in this study represents a dramatic behavioural adaptation by ibis from a transient species moving between ephemeral wetlands (i.e. no site fidelity) to a resident within a relatively constant urban environment. This finding has important implications for the conservation and management of ibis. The high level of site fidelity suggests that birds from colonies occurring at locations distant from priority human assets (e.g. airport flight paths) are unlikely to have a major impact on people. These locations could effectively be managed as refuge habitat [8] and, with the establishment of designated refuges, the management of priority nuisance colonies is less likely to have a serious impact on the regional productivity of urban ibis.

### Levels of Site-fidelity

Birds that were known to be alive but not seen on a particular survey could have been absent for three reasons. Firstly, some birds would have been present in the study site, but unseen by the observer, either because their legs were not visible, or because they were not encountered. Secondly, observations from across the Sydney region indicated that some ibis make foraging trips out of the study site during the day, but return for some part of each day. These birds may have been outside the study site at the time of the survey but still showed site fidelity at the temporal scale of one day [8]. Thirdly, birds may have taken up residence at another site, either temporarily or permanently. By conducting surveys on three consecutive days (robust design, [41]) we were able to estimate the rates of temporary emigration and immigration, distinguishing birds in the third category from the other two sources of error. The model estimated that between each fortnightly set of surveys ∼20% of observed ibis would not be resighted in the next survey (temporary emigration). Similarly, between quarterly surveys ∼33% of the observed ibis would not be resighted in the next survey. Movements out of the study site may be daily foraging or transient movements for extended periods (e.g. months), however our data suggest that birds are likely to return. Evidence for this is provided by the two immigration estimates. For the fortnightly survey ∼90% of the banded ibis that were absent from a survey were unlikely to be resighted in the next fortnightly survey, but for the quarterly surveys this dropped to ∼65%. This suggests that ibis that ended a period of residency [43] were more likely to stay away from the site for a couple of months than for a couple of weeks.

Female ibis showed greater site fidelity than males. A higher proportion of females were resighted each year and they were resighted more frequently than males throughout each year. There was some indication that males visited external breeding colonies more frequently than females, although this result was not significant. These movements throughout the study might be associated with a bird’s assessment of potential reproductive opportunities. Ibis have been observed to engage in polygamy and extra-pair copulation, although breeding pairs that share parental investment equally have been observed to fledge the most offspring [34], [44].

### Use of Alternative Sites

The habitat type where we detected most birds outside the study site was landfills. Landfills represent a major foraging resource for urban ibis [12] and we observed 41% of females and 50% of males foraging within open landfills at least once. There is no doubt that the percentage of ibis observed within landfills is an underestimate [8], [38] as our surveys covered only a fraction of their operating hours, and leg bands are difficult to detect in this circumstance. Substantially higher resighting rates of marked birds have been recorded with patagial tags compared with leg bands [38], [49], including within landfills. The abundant resources within landfills are likely to be fueling increases in the urban ibis population, the extension of the breeding season [12] and the resultant human-wildlife conflicts. Similar observations of the relationship between population increases and conflicts have been made for other nuisance species [45], [50].

### Population Variability

The ibis population within the study site followed seasonal trends of growth and decline in each year of the study, a trend also observed for the regional population [12]. In association with the breeding season we observed an influx of adult ibis probably from within the study region as well as beyond. Towards the end of the breeding season we observed the adult and juvenile population to decline significantly. Again some birds would disperse within the region while it is likely that others left the Sydney region. For example, we have opportunistic observations of ibis (*n* = 17) outside the study region at locations between 50 km and 400 km (mean = 84 km (±23 se)) distant from the study site. Thus, the movement behaviour of individuals has implications for population dynamics at a broader scale [1], [51] and therefore for management and conservation. The ability of ibis to move long distances is well known and band recoveries have identified movements of 1000’s of kilometres by dispersing juveniles [14], [52]. However, we have a limited understanding of where adult ibis disperse and less is known about the site fidelity or natal philopatry of the birds that do disperse. Of particular interest are the possible interactions between the urban and the inland ‘natural’ populations, but to date, no birds have been recorded transitioning between these populations. (The ‘natural’ inland habitats are difficult to access and have a very low human population density; the ibis population estimates are derived from annual aerial surveys [16], [32].)

Ibis are believed to be long-lived (up to 26-years [31]), which is supported by the high rate of adult survival estimated over the four years of this study (females 92% and males 85%). Urban ibis colonies may be subjected to management, primarily by suppressing recruitment through nest and egg destruction [13]. The aim of this management is to reduce human-wildlife conflicts by decreasing the number of nesting birds or deterring nesting entirely. However, the continual suppression of recruitment in urban areas may have long-term implications as the non-urban ibis population has been declining over the last 20-years [32]. Over the last decade there has been an absence of large ‘natural’ breeding events as a direct result of drought [53] and insufficient environmental flows [33], although the urban population has grown over the same period [12]. Importantly, there may be a (presently unknown) time lag between when reproductive failure and a population reduction is evident in the adult population. Therefore, breeding within the urban environment may be important for the persistence of this species.

Currently we lack knowledge of the recruitment rate of ibis fledglings to the reproductive population (third year) in both urban and natural habitats. Ibis achieve good rates of nesting success in urban areas, with the percentage of eggs laid that fledged in two colonies ranging from 25% (*n* = 541) to 40% (*n* = 343, [52]), but comparable data are yet to be measured within the ephemeral inland wetlands (non-urban). It is likely that the breeding success in urban and ‘natural’ environments may differ [10], [54]. The survival rate in the resource-rich urban environment might be expected to be higher than in natural systems, where variable foraging and the distance between resources may negatively affect survival [54]. Conversely, pollutants present in urban foraging sites may result in lower levels of reproductive success [55]. The non-urban rates of survival, fecundity and particularly colonisation by ‘urban’ ibis could have important implications for the longevity of the non-urban ibis population. Thus recruitment and dispersal from the urban environment may be very important, as has been observed for Florida Scrub Jays and American Crows [10], [46].

### Effectiveness of Management

Wildlife management for population [56] and disease control [57], [58] can result in unexpected and potentially undesirable outcomes. Given that collision with ibis presents an unacceptable risk to jet aircraft, management of colonies or birds which traverse aircraft approach paths is necessary to reduce the collision risk. Killing large numbers of adult birds as a solution may have an unacceptable effect on the broader ibis population given the level of population connectivity [8] and the absence of recent breeding events in their traditional habitat [33]. Nest and egg destruction directed towards reducing population size is an inefficient method of effecting local level population reduction because of the high level of population interchange at a regional scale. The level of nest and egg destruction at the regional scale that would be required to achieve appreciable population reduction at a particular local site would have an unacceptable impact on the species, given its current, urban-biased distribution [12], [32]. The best option for managing nuisance colonies is to remove nesting habitat (usually exotic palms) forcing the colony into alternative habitat. This approach, however, depends on the availability of suitable alternate habitat that can support a large colony and that is distant from sensitive areas (e.g. airports). Such habitat exists but there is often resistance from nearby human residents to accept ibis because they perceive ibis to be a global rather than local pest. If ibis are prevented from settling in large areas of suitable habitat, colonies are likely to fracture and nest in multiple locations near to those from which nesting habitat has been removed. Not only will this fail to alleviate the risk to aircraft, but it is likely to create additional problems with humans. Identifying areas within a region in which ibis will be free from persecution is therefore a fundamental component of ibis management. In the longer term, reduction of food availability at landfills is likely to make urban environments less attractive and may encourage ibis to settle in areas further from human habitation [8].
